# Modeling and Analysis of Wide Frequency Band Coaxial TSV Transmission Interconnect

**DOI:** 10.3390/mi15091127

**Published:** 2024-09-03

**Authors:** Yujie Zhang, Changle Zhi, Gang Dong

**Affiliations:** School of Microelectronics, Xidian University, Xi’an 710000, China; 18291602215@163.com (Y.Z.); zhichangle@163.com (C.Z.)

**Keywords:** CTSV interconnect, CTSV, RDL, bump, equivalent circuit model, *S*-parameter, parametric analysis

## Abstract

In this paper, we first build the 3D model of coaxial TSV(CTSV), RDL, and bump of the CTSV interconnect, and extract the equivalent circuit model of each part. Then, we get the *S*-parameters of the 3D and equivalent circuit model of the CTSV interconnect structure; the validity of the equivalent circuit model is verified by comparing the consistency of the *S*-parameters. The simulation results show that the maximum errors for the *S*_11_ and *S*_21_ parameters are 0.4% and 0.18%, respectively, which proves the validity of the equivalent circuit modeling in this paper. Finally, parametric analysis is performed to investigate the effect of different model parameters on the signal-transmission characteristics of the CTSV interconnect.

## 1. Introduction

At present, as the feature size continues to shrink and the package integration continues to improve, the interconnect density within the terahertz system has approached the limit of the current stage of the process, the negative impact of electromagnetic coupling and crosstalk has become more and more significant, and traditional through-Silicon-Via (TSV) can no longer satisfy the needs. Therefore, coaxial TSV(CTSV) with redistribution layer (RDL) and bump process has received widespread attention from industry and academia [1,2,3,4,5,6,7,8,9,10,11,12]. The special 3D coaxial structure embedded in the substrate can effectively shield the transmission path of terahertz band signals while maintaining a very high level of integration and reduce the signal crosstalk and parasitic loss between the interconnection paths [13,14,15]. At the same time, the interconnection system of CTSV with RDL and bump can be compatible with the requirements of interconnection applications within terahertz integrated systems and is gradually becoming the first choice for high-density microsystem integration [16,17]. Currently, there are some modeling research works on CTSV [18,19,20]. In [17], the electrical performance of CTSV is examined. The full-wave extraction and empirical calculations show good agreement in TSV passive elements (RLGC). In [19], based on the quasi-magnetostatic theory, the equivalent electrical parameters of silicon-core CTSVs is extracted, and the corresponding distributed transmission line model is introduced. The proposed model is validated against the 3D full-wave field solver. In [20], silicon-core CTSVs are modeled and studied, in which the inner metal via is replaced with a Cu-coated silicon pole. Results show the proposed CTSVs have comparable performance to standard Cu-based CTSVs. However, there are some shortcomings of the existing studies. To begin with, the main research at present is the single CTSV, not the CTSV interconnect containing CTSV, RDL, and bump, which does not match the actual 3D circuit structure. For another, the operating frequency of existing research is low, which cannot be used in high-frequency scenes. Moreover, the influence of different parameters on signal-transmission characteristics of CTSV interconnect is not researched, so what influences the transmission characteristics of CTSV interconnect is unknown.

In this work, we propose a modeling method that accurately models CTSV, RDL, and bump of CTSV interconnect operating at frequencies exceeding 100 GHz. In Section 2, we establish the 3D structure model and equivalent circuit models of the CTSV, the RDL, and the bump, and extract the electrical parameters of the models. The simulations are carried out in Section 3; the *S*-parameters are utilized to validate the validity of the proposed CTSV interconnect equivalent electrical model. When comparing *S*-parameters, we subtract the *S*-parameter data in the structural model from the corresponding data in the circuit model to create an error curve graph. Based on the results in the graph, we ensure the consistency of the corresponding *S*-parameters between the two and verify the accuracy of the *S*-parameter comparison. Parametric scan is performed in Section 4 to research the influence of different parameters on the signal-transmission characteristics of the CTSV interconnect. Finally, conclusions are drawn in Section 5.

Compared to the results of previous studies, the contributions of this paper are as follows:

(1) Accurate equivalent circuit model containing CTSV, RDL, and bump is built.
(2) Due to the accuracy of distributed parameter circuits at high frequencies [21], the equivalent circuit model can perfectly match the actual situation more than 100 GHz.
(3) The influence of different parameters on transmission characteristics of the CTSV interconnect is studied, providing a basis for the CTSV interconnect design.

## 2. 3D Structural Modeling and Equivalent Circuit Extraction of the CTSV Interconnect

**Figure 1:**
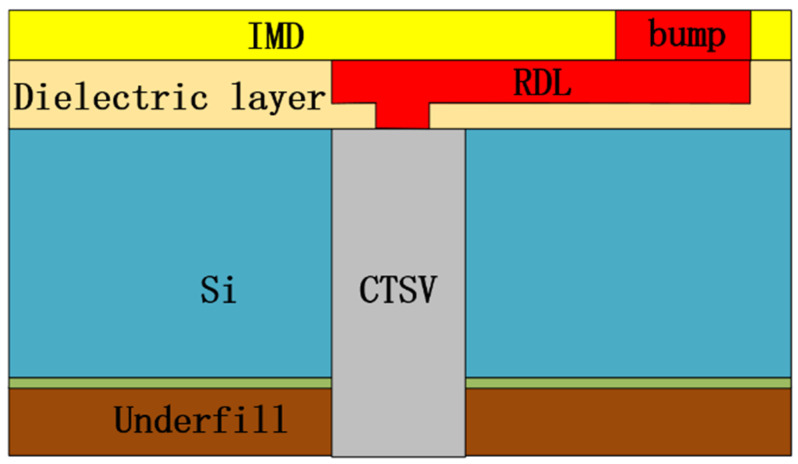
Structural diagram of the built CTSV interconnect.

**Figure 2:**
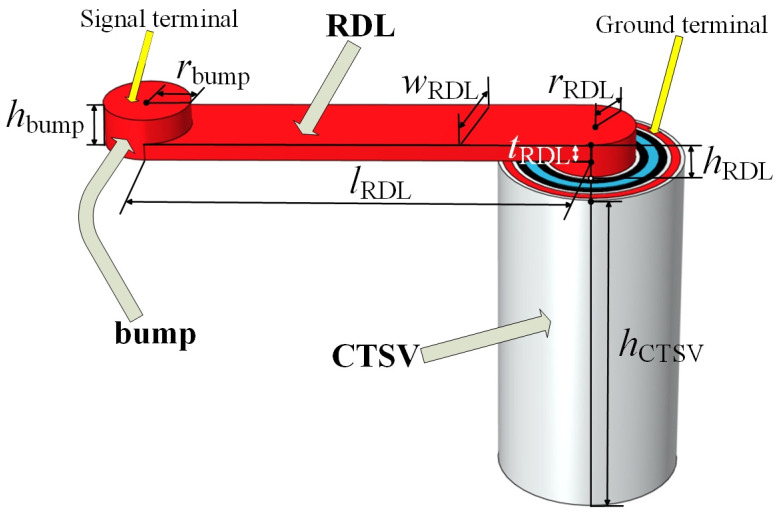
3D view of the interconnect and related dimensions.

**Figure 3:**
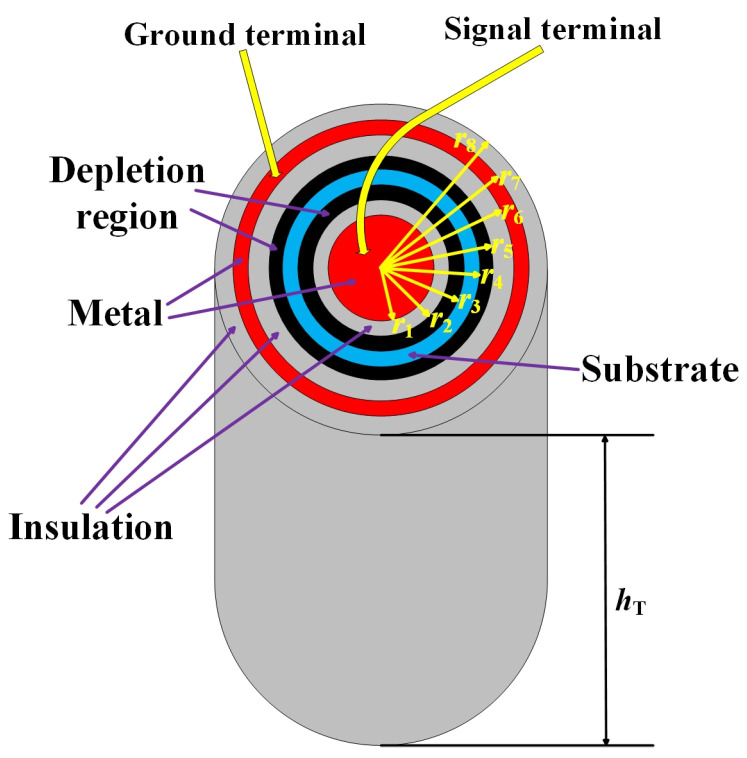
3D view of the CTSV and related dimensions.

The structural diagram of the CTSV interconnect we build is shown in Figure 1, which contains CTSV, RDL, and bump, in which RDL and bump are wrapped by BCB. The 3D view of the interconnect and the CTSV are shown in Figure 2 and Figure 3, the values of relevant model parameters of the CTSV interconnect are shown in Table 1, and the electrical parameters are selected based on the specific values of various materials at room temperature. It can be seen that for the CTSV in Figure 3, the inner metal, inner insulation, and Si substrate form a MOS structure. And at the same time, the external metal, middle insulation, and Si substrate form a MOS structure. Each MOS structure generates a depletion region. As a result, the depletion regions are formed at two locations [22].

In Figure 3, there is an internal depletion region, an external depletion region, and a Si substrate in Si region. For most applications, the Si substrate can be seen as the ground, so the potential of which is probably zero. For the depletion regions, the potential of which can be calculated using the Poisson equation in the cylindrical coordinate system. Because the middle silicon substrate can be seen as the ground, the potential and electric field at the boundary of the silicon substrate are both zero [12,23]. The boundary conditions are shown in Equations (1) and (2).

(1) $$\frac{1}{r}\frac{d}{dr}(r\frac{d\varphi }{dr})=\frac{{\mathrm{qN}}_{a}}{\varepsilon_{\mathrm{Si}}}$$

(2) $$\;\varphi {|}_{{r=r}_{i}}=0,\frac{d\varphi }{dr}{|}_{{r=r}_{i}}=0,\;i=3,4$$

By integrating Equations (1) and (2), the potentials of the depletion regions can be obtained, which are shown in Equations (3) and (4).

(3) $$\varphi_{\mathrm{dep},\mathrm{in}}=\frac{{\mathrm{qN}}_{a}}{2\varepsilon_{\mathrm{Si}}}\left(\frac{r_{2}^{2}-r_{3}^{2}}{2}-r_{3}^{2}\cdot \ln\frac{r_{2}}{r_{3}}\right)$$

(4) $$\varphi_{\mathrm{dep},\mathrm{in}}=\frac{{\mathrm{qN}}_{a}}{2\varepsilon_{\mathrm{Si}}}\left(\frac{r_{5}^{2}-r_{4}^{2}}{2}-r_{4}^{2}\cdot \ln\frac{r_{5}}{r_{4}}\right)\;$$

For the outer metal of CTSV, it is grounded so the potential is zero. However, for the inner metal, the upper surface is the input port, whose potential is determined by an external input signal [22].

### 2.1. Equivalent Circuit Extraction of the CTSV

**Figure 4:**
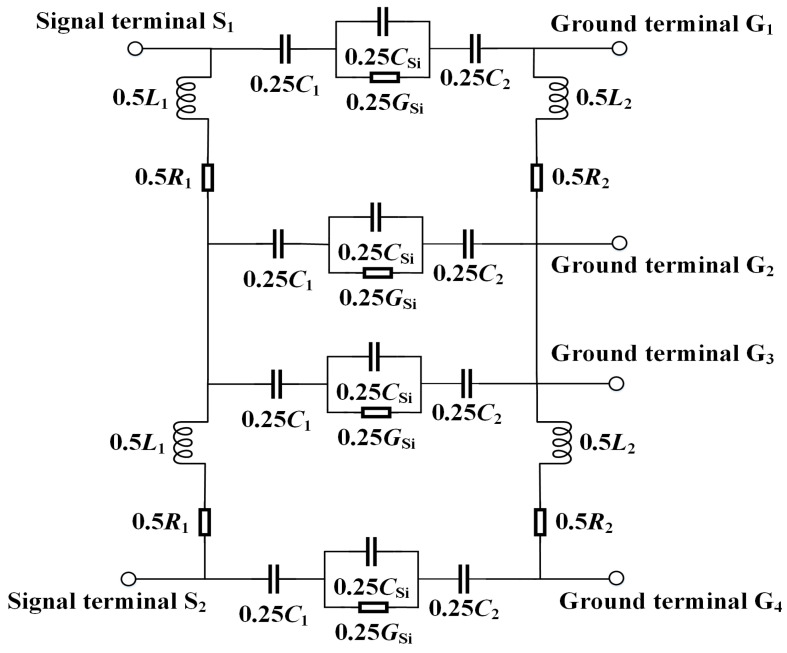
Proposed equivalent circuit model of the CTSV.

The proposed equivalent circuit model of the CTSV is shown in Figure 4. *C*_Si_ characterizes the influence of intermediate silicon substrate capacitance, *G*_Si_ characterizes the influence of intermediate silicon substrate conductance, *C*_ox1_ characterizes the influence of internal oxide layer capacitance, *C*_ox2_ characterizes the influence of external oxide layer capacitance, *C*_d1_ characterizes the influence of internal depletion layer capacitance, *C*_d2_ characterizes the influence of external depletion layer capacitance, *C*_1_ characterizes the series connection of internal oxide capacitance and internal depletion layer capacitance, *C*_2_ characterizes the series connection of external oxide capacitance and external depletion layer capacitance, δ characterizes skin depth, *L*_1_ and *R*_1_ characterize the influence of internal metal conductor, and *L*_2_ and *R*_2_ characterize the influence of external shielding layer metal.

In modern physics, there is a theory of using metal shells to achieve electrostatic shielding, which can also be extended to the three-dimensional structure of CTSVs. Although the external metal does not completely wrap around the inner copper shaft, the inner copper is a signal-transmission line, and the upper and lower ends are also connected by metal. The signal influence of its upper and lower ports can be ignored. From this perspective, it can be considered that the inner copper that transmits signals is still completely isolated by the external metal. Therefore, according to the theory of electrostatic shielding, the influence of the external metal structure can be ignored. As a layer that isolates interference, the thickness of the outer metal will not affect its anti-interference ability. Even in different environments with significant external interference, this shielding effect still applies, and the influence of external metal structures can still be ignored [12,24]. Similar to [24], Equations (5) to (21) are analytic expressions of the electrical parameters of the CTSV equivalent circuit model. *C*_Si_, *G*_Si_, *C*_ox1_, *C*_ox2_, *C*_d1_, *C*_d2_, *C*_1_, and *C*_2_ are shown from Equations (5) to (12).

(5) $$C_{\mathrm{Si}}=\frac{{2\pi \varepsilon }_{\mathrm{Si}}h_{\mathrm{CTSV}}}{\ln(\frac{r_{4}}{r_{3}})}$$

(6) $$G_{\mathrm{Si}}=\frac{C_{\mathrm{Si}}\sigma_{\mathrm{Si}}}{\varepsilon_{\mathrm{Si}}}$$

(7) $$C_{\mathrm{ox}1}=\frac{{2\pi \varepsilon }_{\mathrm{ox}}h_{\mathrm{CTSV}}}{\ln(\frac{r_{2}}{r_{1}})}$$

(8) $$C_{\mathrm{ox}2}=\frac{{2\pi \varepsilon }_{\mathrm{ox}}h_{\mathrm{CTSV}}}{\ln(\frac{r_{6}}{r_{5}})}$$

(9) $$C_{d1}=\frac{{2\pi \varepsilon }_{d}h_{\mathrm{CTSV}}}{\ln(\frac{r_{3}}{r_{2}})}$$

(10) $$C_{d2}=\frac{{2\pi \varepsilon }_{d}h_{\mathrm{CTSV}}}{\ln(\frac{r_{5}}{r_{4}})}$$

(11) $$C_{1}=(\frac{1}{C_{\mathrm{ox}1}}+\frac{1}{C_{d1}}{)}^{-1}$$

(12) $$C_{2}=(\frac{1}{C_{\mathrm{ox}2}}+\frac{1}{C_{d2}}{)}^{-1}$$

*L*_1_ and *L*_2_ are shown from Equations (13) and (14).

(13) $$L_{1}=\frac{{\mu h}_{\mathrm{CTSV}}}{2\pi }\cdot \ln(\frac{r_{3}}{r_{1}})$$

(14) $$L_{2}=\frac{{\mu h}_{\mathrm{CTSV}}}{2\pi }\cdot \ln(\frac{r_{6}}{r_{4}})$$

δ and expressions of relevant resistors are shown from Equations (15) to (21).

(15) $$\delta =\sqrt{\frac{2}{{\omega \mu \sigma }_{\mathrm{Cu}}}}$$

(16) $$R_{1,\mathrm{dc}}=\rho \cdot \frac{h_{\mathrm{CTSV}}}{{\pi \cdot r}_{1}^{2}}$$

(17) $$R_{1,\mathrm{ac}}=\rho \cdot \frac{h_{\mathrm{CTSV}}}{{\pi \cdot [r}_{1}^{2}-{\left(r_{1}-\delta \right)}^{2}]}$$

(18) $$R_{1}=\sqrt{R_{1,\mathrm{dc}}^{2}{+R}_{1,\mathrm{ac}}^{2}}$$

(19) $$R_{2,\mathrm{dc}}=\rho \cdot \frac{h_{\mathrm{CTSV}}}{{\pi \cdot (r}_{7}^{2}-r_{6}^{2})}$$

(20) $$R_{2,\mathrm{ac}}=\rho \cdot \frac{h_{\mathrm{CTSV}}}{{\pi \cdot [r}_{7}^{2}-{\left(r_{7}-\delta \right)}^{2}]}$$

(21) $$R_{2}=\sqrt{R_{2,\mathrm{dc}}^{2}{+R}_{2,\mathrm{ac}}^{2}}$$

### 2.2. Equivalent Circuit Extraction of the RDL

**Figure 5:**
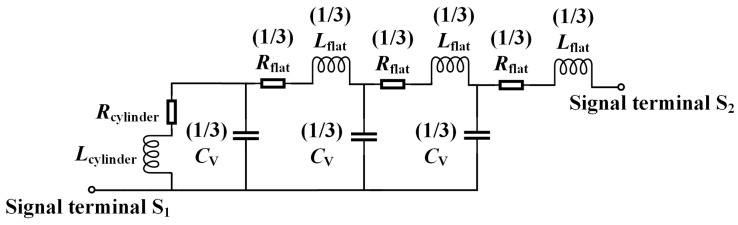
Proposed equivalent circuit model of the RDL.

In practical CTSV interconnect, redistribution layer (RDL) can provide a horizontal connection path between CTSV and bump to redistribute signals between heterogeneous chips. The proposed equivalent circuit model of the RDL is shown in Figure 5, in which *R*_cylinder_ and *L*_cylinder_ represent the influence of the cylindrical metal in the vertical direction of the RDL, *R*_flat_ and *L*_flat_ represent the influence of the square metal in the horizontal direction of the RDL, and *C*_V_ characterizes the capacitance effect due to the presence of the BCB insulation layer between the square metal plane of the RDL and the conductive part of the CTSV.

*R*_cylinder_, *L*_cylinder_, *R*_flat_, *L*_flat_, and *C*_V_ are shown from Equations (22) to (30).

(22) $$R_{\mathrm{cylinder},\mathrm{dc}\;}=\rho \cdot \frac{h_{\mathrm{RDL}}}{{\pi \cdot r}_{\mathrm{RDL}}^{2}}\;\;\;$$

(23) $$R_{\mathrm{cylinder},\mathrm{ac}}=\rho \cdot \frac{h_{\mathrm{RDL}}}{{\pi \cdot [r}_{\mathrm{RDL}}^{2}-{\left(r_{\mathrm{RDL}}-\delta \right)}^{2}]}$$

(24) $$R_{\mathrm{cylinder}}=\sqrt{R_{\mathrm{cylinder},\mathrm{dc}}^{2}{+R}_{\mathrm{cylinder},\mathrm{ac}}^{2}}$$

(25) $$L_{\mathrm{cylinder}}{=2h}_{\mathrm{RDL}}\cdot (\ln\frac{4h_{\mathrm{RDL}}}{r_{\mathrm{RDL}}}-0.75)$$

(26) $$R_{\mathrm{flat},\mathrm{dc}}=\rho \cdot \frac{l_{\mathrm{RDL}}}{t_{\mathrm{RDL}}{\cdot w}_{\mathrm{RDL}}}$$

(27) $$R_{\mathrm{flat},\mathrm{ac}}=\rho \cdot \frac{l_{\mathrm{RDL}}}{{(t}_{\mathrm{RDL}}{\cdot w}_{\mathrm{RDL}}{)-[(t}_{\mathrm{RDL}}-\delta )\cdot \left(w_{\mathrm{RDL}}-\delta \right)]}$$

(28) $$R_{\mathrm{flat}}=\sqrt{R_{\mathrm{flat},\mathrm{dc}}^{2}{+R}_{\mathrm{flat},\mathrm{ac}}^{2}}$$

(29) $$L_{\mathrm{flat}}\;=2l_{\mathrm{RDL}}\cdot (\ln\frac{2l_{\mathrm{RDL}}}{w_{\mathrm{RDL}}}+0.5+0.2235\frac{w_{\mathrm{RDL}}}{l_{\mathrm{RDL}}})$$

(30) $$Cv=\frac{\varepsilon_{\mathrm{BCD}}}{d_{\mathrm{RDL}}}\cdot S_{\mathrm{RDL}}$$

### 2.3. Equivalent Circuit Extraction of the Bump

**Figure 6:**
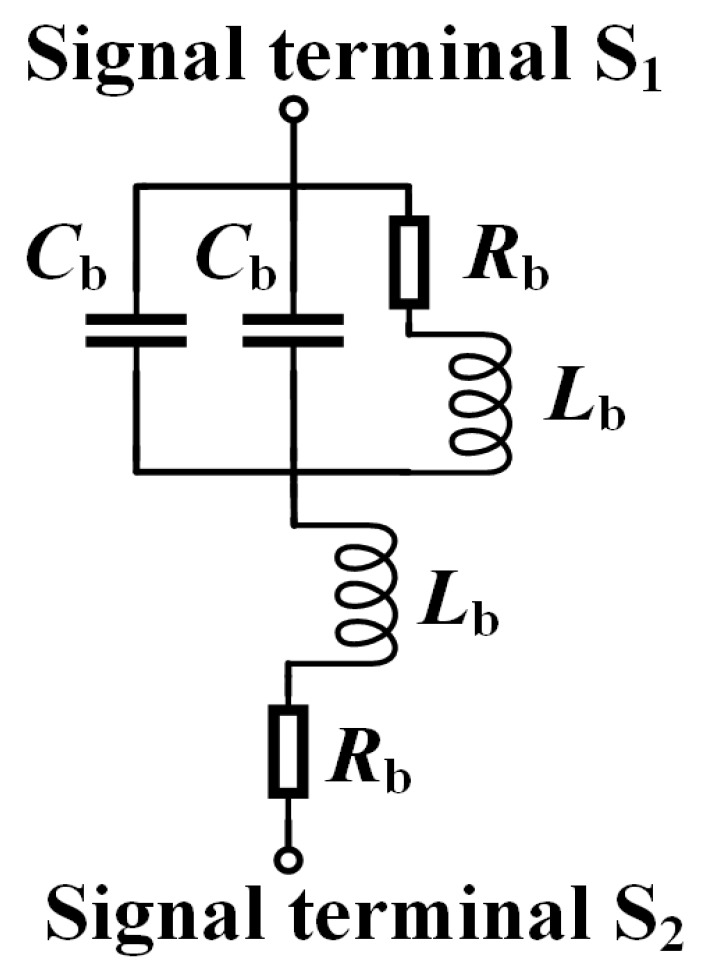
Proposed equivalent circuit model of the bump.

In practical CTSV interconnect, bump can provide vertical connections and stress buffering of the chips, and to transmit signals from CTSV to the next layer of chips via RDL. The proposed equivalent circuit of the bump is shown in Figure 6, in which *R*_b_ and *L*_b_ characterize the effect of the metal cylinder part, and *C*_b_ characterizes the capacitive effect between the metal cylinder and adjacent conductor of the RDL.

*R*_b_, *L*_b_ and *C*_b_ are shown from Equations (31) to (35).

(31) $$R_{b,\mathrm{dc}\;}=\rho \cdot \frac{h_{\mathrm{bump}}}{{\pi \cdot r}_{\mathrm{bump}}^{2}}$$

(32) $${\;R}_{b,\mathrm{ac}}=\rho \cdot \frac{h_{\mathrm{bump}}}{{\pi \cdot [r}_{\mathrm{bump}}^{2}-{\left(r_{\mathrm{bump}}-\delta \right)}^{2}]}$$

(33) $$R_{b}=\sqrt{R_{b,\mathrm{dc}}^{2}{+R}_{b,\mathrm{ac}}^{2}}$$

(34) $$L_{b}{=2h}_{\mathrm{bump}}\cdot (\ln\frac{4h_{\mathrm{bump}}}{r_{\mathrm{bump}}}-0.75)$$

(35) $$C_{b}=\frac{\varepsilon_{\mathrm{BCD}}}{d_{\mathrm{bump}}}\cdot S_{\mathrm{bump}}$$

## 3. Simulation and Verification

**Figure 7:**
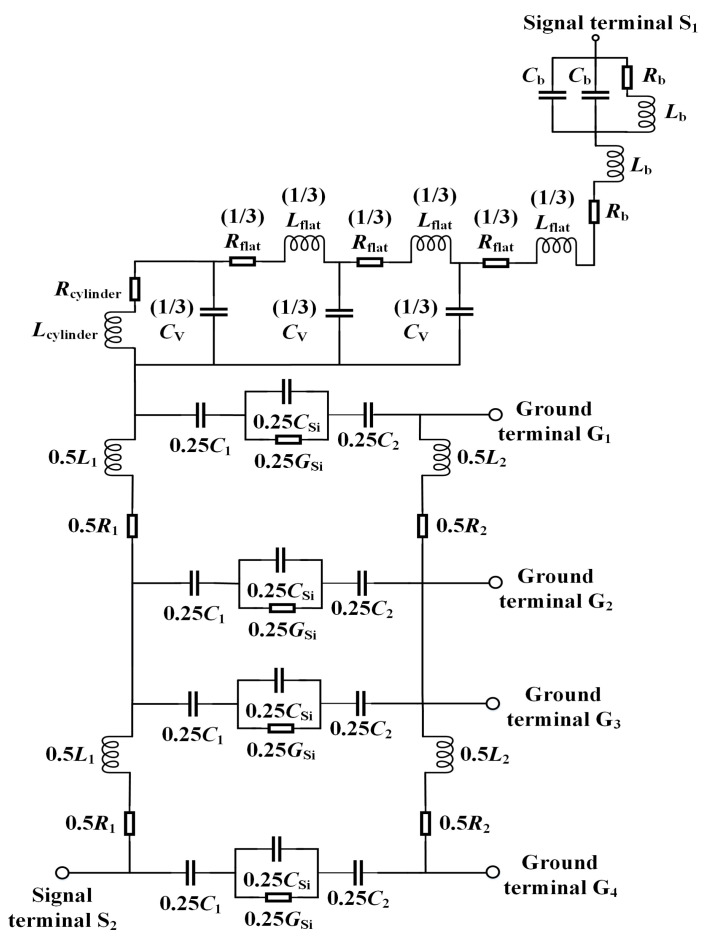
Proposed equivalent circuit model of the complete CTSV interconnect.

**Figure 8:**
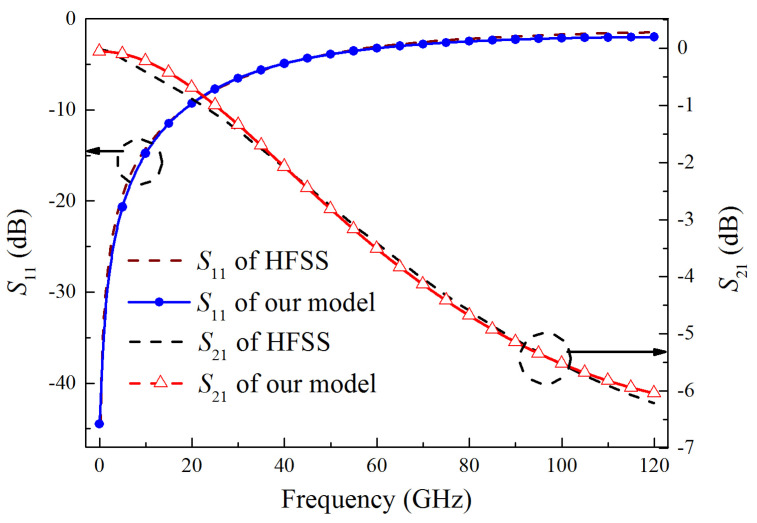
Comparison of simulation results of *S*_11_ and *S*_21_ of the CTSV interconnect.

In order to verify the validity of the established equivalent circuit model, the 3D structural model in HFSS (Version: 2022 R1) and the equivalent circuit model in ADS (Version: 2022) are simulated up to 120 GHz to obtain their *S*-parameters. The high consistency in simulation accuracy and results between HFSS and ADS simulation software has been verified in literature [5]. In the structural model of HFSS and the circuit model of ADS, the input-output ports are corresponded to the same, the excitations of the two input ports are set to be the same, and the grounding regions are also set to be the same. These operations ensure consistency between the two simulation environments. Relevant parameters of the model are shown in Table 1. The equivalent circuit model of the complete CTSV interconnect is shown in Figure 7, and the simulation results are shown in Figure 8. As can be seen from Figure 8, the *S*_11_ curves of the 3D model and the equivalent circuit model are in perfect agreement in the frequency range from 0.1 to 120 GHz, and the simulation error is basically no more than 1%. The *S*_21_ curves agree better, especially around the desired 100 GHz frequency, in which the maximum error is no more than 0.48%. These errors mainly come from parasitic resistance, capacitance, and inductance, among which the errors caused by parasitic capacitance are obvious at low frequencies, the errors caused by parasitic inductance are obvious at high frequencies, and the contributions of parasitic resistance to the errors are almost the same at different frequencies [13]. In order to reduce these errors, we adopt the distributed parameter method when establishing the equivalent circuit model and consider parasitic effects into the circuit model. We compare the simulation results of the obtained circuit model with those in HFSS, analyze the reasons for the differences between the two at different frequencies, continuously optimize the obtained circuit model, and finally obtain an equivalent circuit model with the minimum error.

The results of Figure 8 demonstrate the validity of the equivalent circuit model of the CTSV interconnect. Compared with the results in [25], the model in this paper not only contains all parts of the CTSV interconnect, including CTSV, RDL, and bump, but also has more accurate results. Furthermore, the time spent on simulation with the equivalent circuit model is significantly reduced compared with full-wave simulation software. Due to the consideration of various non-ideal effects and the use of more accurate distributed parameter models, our model has good scalability even for larger and more complex interconnect structures. Although we do not operate actual experimental measurements on the CTSV interconnect model established in the article, the results obtained by HFSS are almost identical to the actual experimental results [12,26].

## 4. Parametric Analysis

In order to research the influence of different parameters on the signal-transmission characteristics of the CTSV interconnect, in this section, we perform parametric scan. For CTSV, the height and internal metal radius will affect the signal-transmission characteristics, but changing the internal metal radius also changes the size of the RDL. For the RDL, its length, width, and thickness all have an impact on signal-transmission characteristics. However, the length of the RDL is determined by the actual interconnect structure rather than the designer, so only the width and thickness of the RDL are analyzed as parameters. For bump, its height and radius can affect the signal-transmission characteristics. But the radius of the bump is determined by the RDL, so only its height will be analyzed as a parameter [26]. Taking these factors into consideration, we operate parameter analysis on CTSV height, RDL width and thickness, and bump height that can be independently set, but do not analyze the remaining parameters that cannot be independently set. Except for the parameters that need to be scanned, the other parameters are shown in Table 1.

### 4.1. Scanning Analysis of CTSV Height

**Figure 9:**
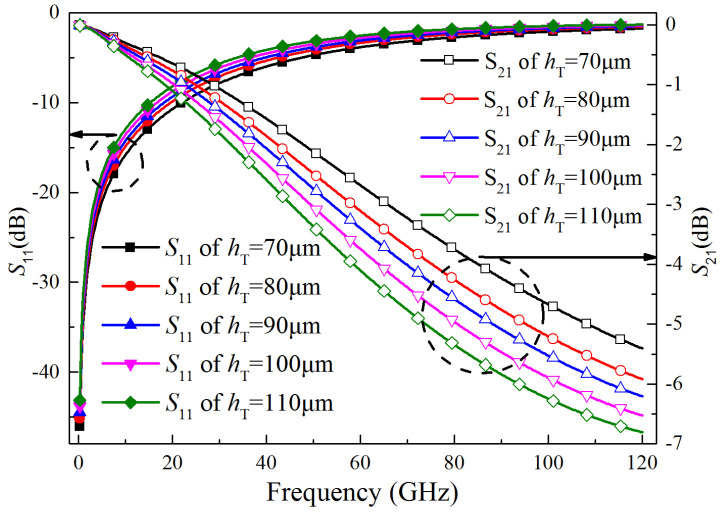
*S*_11_ and *S*_21_ of CTSV interconnect at different heights.

The CTSV height scanning analysis results are shown in Figure 9. As can be seen from Figure 9, *S*_11_ increases and *S*_21_ decreases with the increasing CTSV height. The reason is that as the CTSV height increases, the signal-transmission distance becomes larger and the associated parasitic parameters become larger, especially the effect of parasitic capacitance. The larger parasitic parameters make the attenuation of the signal increase and the transmission quality decrease, so small CTSV height is more favorable for the signal transmission. At the same time, however, from the process point of view, a small CTSV height will reduce the depth-to-width ratio of the CTSV, increase the difficulty of production, and decrease the distance between different chips, which is likely to occur in coupling.

### 4.2. Scanning Analysis of RDL Width and Thickness

**Figure 10:**
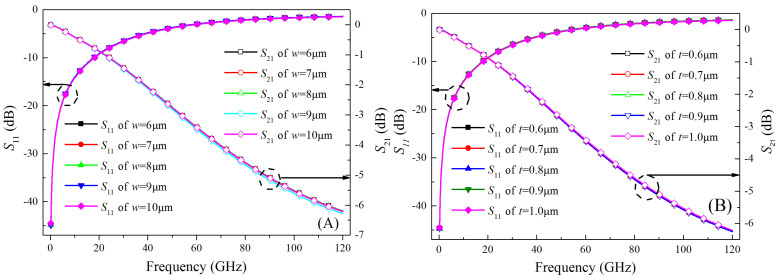
Comparison of simulation results of *S*_11_ and *S*_21_ of the CTSV interconnect at different widths (**A**) and thicknesses (**B**).

The RDL width and thickness scanning analysis results are shown in Figure 10. From the results of Figure 10, we can see that neither the width nor the thickness of the RDL have a significant effect on the signal-transmission characteristics of the CTSV interconnect. This is due to the fact that the factors affecting the transmission characteristics of the RDL are primarily the RDL length, which is determined by practical interconnect structure, rather than RDL width and thickness, which are determined by the designer. Therefore, the changes in width and thickness of the RDL do not have a great effect on its transmission characteristics.

### 4.3. Scanning Analysis of Bump Height

**Figure 11:**
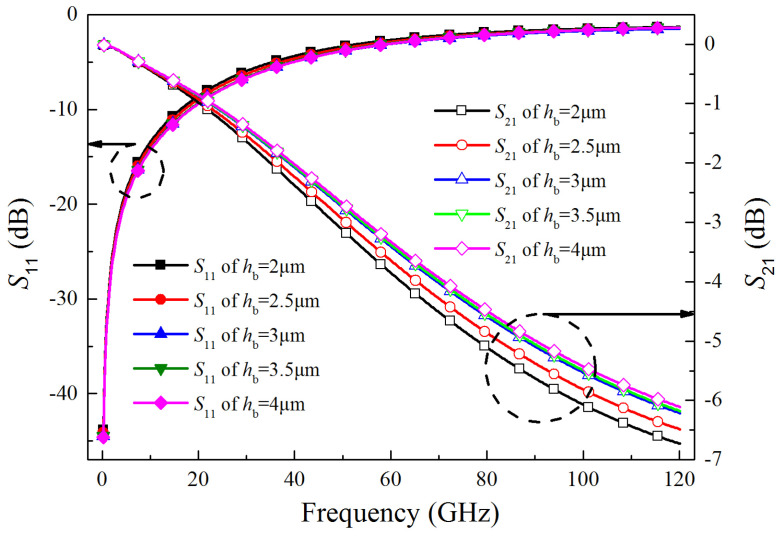
*S*_11_ and *S*_21_ of CTSV interconnect at different bump heights.

The bump height scanning analysis results are shown in Figure 11. From Figure 11, S_11_ decreases slightly with the increase of bump height, and within a certain range, S_21_ increases with the increase of bump height, which indicates that the signal-transmission performance becomes better as the bump height increases. But this effect is no longer significant when the bump height is greater than 3.5 μm. The reason of which is related to the impedance matching of the bump signal port.

## 5. Conclusions

In this paper, we firstly build a 3D structural model of the CTSV interconnect and build the equivalent circuit models of each part according to the 3D model and extract the electrical parameters. Then, the 3D structure model and its equivalent circuit model are simulated in the frequency range from 0.1 to 120 GHz. The simulation results show that the S11 and S21 parameters of the equivalent circuit model are in good agreement with the 3D structure model with very small error. Finally, parameter scan is carried out to investigate the effect of different parameters on the signal-transmission performance of the CTSV interconnect. The results of this paper can provide guidance for the equivalent circuit modeling and parameter analysis of CTSV interconnect in terahertz wide frequency band.

## Figures and Tables

**Table 1:** Relevant model parameters of the CTSV interconnect.

Parameter	Symbol/Unit	Value
CTSV height	*h*_CTSV_/μm	90
Inner Cu cylinder radius of the CTSV	*r*_1_/μm	5
Outside diameter of internal insulation of the CTSV	*r*_2_/μm	5.5
Outside diameter of internal depletion region of the CTSV	*r*_3_/μm	6.4
Outside diameter of Si substrate of the CTSV	*r*_4_/μm	7.6
Outside diameter of external depletion region of the CTSV	*r*_5_/μm	8.5
Outside diameter of middle insulation of the CTSV	*r*_6_/μm	9
Outside diameter of external Cu cylinder of the CTSV	*r*_7_/μm	10
Outside diameter of external insulation of the CTSV	*r*_8_/μm	10.5
Metal cylindrical radius of the RDL	*r*_RDL_/μm	5
Metal cylindrical height of the RDL	*h*_RDL_/μm	3
Metal flat length of the RDL	*l*_RDL_/μm	100
Metal flat width of the RDL	*w*_RDL_/μm	10
Metal flat thickness of the RDL	*t*_RDL_/μm	1
Distance between RDL and CTSV	*d*_RDL_/μm	3
Area between the RDL metal flat and the CTSV conductor section	*S*_RDL_/μm^2^	28.36
Metal cylindrical radius of the bump	*r*_bump_/μm	5
Metal cylindrical height of the bump	*h*_bump_/μm	3
Equivalent distance between the bump metal cylinder and adjacent conductor	*d*_bump_/μm	1.8
Equivalent area between the bump metal cylinder and adjacent conductor	*S*_bump_/μm^2^	15.7
Relative permittivity of oxide	ε_ox_/1	4
Relative permittivity of Si	ε_Si_/1	11.9
Relative permittivity of depletion region	ε_ox_/1	11.9
Conductivity of Si	σ_Si_/(S/m)	7.1
Conductivity of Cu	σ_Cu_/(S/m)	5.8 × 10^7^
Resistivity of Cu	ρ/(Ω·m)	1.8 × 10^−8^
Relative permeability of Cu	μ/1	9.999 × 10^−1^
Relative permittivity of BCD	ε_BCD_/1	2.6
Operating angle frequency	*ω*/(rad/s)	200π

## Data Availability

The data used to support the findings of this study are included within the article.
